# Investigating socioeconomic disparities in lung cancer diagnosis, treatment and mortality: an Italian cohort study

**DOI:** 10.1186/s12889-024-19041-4

**Published:** 2024-06-07

**Authors:** Michela Servadio, Alessandro C. Rosa, Antonio Addis, Ursula Kirchmayer, Ilaria Cozzi, Paola Michelozzi, Riccardo Cipelli, Franca Heiman, Marina Davoli, Valeria Belleudi

**Affiliations:** 1 Department of Epidemiology, Regional Health Service Lazio, Rome, Italy; 2 IQVIA Solutions Srl, Milan, Italy

**Keywords:** Lung cancer, Socioeconomic disparities, Diagnosis, Treatment, Mortality

## Abstract

**Background:**

Lung cancer is one of the most lethal cancers worldwide and patient clinical outcomes seem influenced by their socioeconomic position (SEP). Since little has been investigated on this topic in the Italian context, our aim was to investigate the role of SEP in the care pathway of lung cancer patients in terms of diagnosis, treatment and mortality.

**Methods:**

This observational retrospective cohort study included patients discharged in the Lazio Region with a lung cancer diagnosis between 2014 and 2017. In the main analysis, educational level was used as SEP measure. Multivariate models, adjusted for demographic and clinical variables, were applied to evaluate the association between SEP and study outcomes, stratified for metastatic (M) and non-metastatic (NM) cancer. We defined a diagnosis as 'delayed' when patients received their initial cancer diagnosis after an emergency department admission. Access to advanced lung cancer treatments (high-cost, novel and innovative treatments) and mortality were investigated within the 24-month period post-diagnosis. Moreover, two additional indicators of SEP were examined in the sensitivity analysis: one focusing on area deprivation and the other on income-based exemption.

**Results:**

A total of 13,251 patients were identified (37.3% with metastasis). The majority were males (> 60%) and over half were older than 70 years. The distribution of SEP levels among patients was as follow: 31% low, 29% medium–low, 32% medium–high and 7% high. As SEP increased, the risks of receiving a delayed diagnosis ((high vs low: M: OR = 0.29 (0.23–0.38), NM: OR = 0.20 (0.16–0.25)) and of mortality ((high vs low M: OR = 0.77 (0.68–0.88) and NM: 0.61 (0.54–0.69)) decreased. Access to advanced lung cancer treatments increased in accordance with SEP only in the M cohort (high vs low: M: OR = 1.57 (1.18–2.09)). The primary findings were corroborated by sensitivity analysis.

**Conclusions:**

Our study highlighted the need of public health preventive and educational programs in Italy, a country where the care pathway of lung cancer patients, especially in terms of diagnosis and mortality, appears to be negatively affected by SEP level.

**Supplementary Information:**

The online version contains supplementary material available at 10.1186/s12889-024-19041-4.

## Introduction

Over the past three decades, cancer has remained one of the leading causes of death among adults worldwide. Among various types of cancer, lung cancer stands out as the deadliest and most commonly diagnosed form [1, 2]. In Italy, estimates for 2022 indicated a total of 43,900 new cases of lung cancer, with 5-year survival rates around 16% for men and 23% for women [3].

Numerous factors contribute to the high mortality associated with lung cancer. Patients are generally diagnosed at a later stage, because early-stage lung cancer is often associated with a lack of overt symptoms while the detection and interpretation of symptoms require multiple procedures or specialist visits, further delaying diagnosis [4]. In addition, although low-dose computed tomography screening could substantially reduce mortality rates [5–7], its implementation is hindered by concerns over cost-effectiveness, complexity, target population selection and participation rates [8]. Additionally, the exposure to many environmental factors can increase the risk of developing lung cancer, especially smoking, which is thought to be the cause of around 90% of lung cancer diagnoses [9] and also radon, air pollution and asbestos [10]. Moreover, lung cancer predominantly affects older adults, with an average diagnosis age of 70 years [11], indicating increased vulnerability compared to patients with other cancer types.

Regardless of the specific disease, socioeconomic factors, such as income, education, and occupation are considered proxies for underlying determinants of health, referred to as “causes of the causes” by Braveman and Gottieb [12]. Indeed, socially deprived individuals use healthcare services less frequently, have limited access to specialty care, and increased exposure to environmental risk factors (including asbestos, industrial waste, air pollution), compared to those with higher Socioeconomic Position (SEP) [12]. Furthermore, socioeconomic factors may influence access to healthcare services throughout individuals' lifespan, leading to worsened health outcomes in adulthood among more disadvantaged people [13].

In this context, previous studies have demonstrated how socioeconomic and racial disparities affect screening eligibility and utilization [14, 15], as well as cancer incidence and mortality rates [16].

Despite universal healthcare coverage in Italy, socioeconomic disparities persist, affecting cancer risk factor distribution and adherence to screening programs [17]. However, limited research has explored the impact of socioeconomic inequalities on clinical outcomes among lung cancer patients.

In this context, understanding whether socioeconomic indicators in Italy play a role in diagnosis, treatment and mortality of lung cancer patients may help to improve clinical outcomes for this highly deadly type of cancer. Moreover, considering the impact of the COVID pandemic on delays in cancer healthcare management, including screening, prevention and treatment delivery [18, 19], exploring socioeconomic disparities among lung cancer patients in Italy, prior to the onset of pandemic, becomes even more important.

For these reasons, our study aimed to investigate potential associations between the healthcare management of lung cancer patients and indicators of socioeconomic inequalities, at both individual and area level, using real world data from Italian health administrative databases.

## Material and methods

### Study design, data source, and population

The data used for our observational retrospective cohort study were extracted from health administrative databases of the Lazio, a central region of Italy, covering almost 5.8 million inhabitants. Specifically, we sourced data from various databases: the Healthcare Assistance Registry (HAR), which record details of enrolment in the regional healthcare system such as date of birth, sex and registration date; the Hospital Information System (HIS) that collects information on all hospital discharges within regional hospitals, including admission and discharge dates, as well as primary and secondary diagnoses and procedures coded according to the International Classification of Diseases, Ninth Revision, Clinical Modification (ICD-9-CM); the Outpatient Specialist Care database, which contains information on all specialized outpatient services provide within the National Healthcare Services (NHS); the Drug Dispensing Registry, collecting information on medications dispensed through facilities not directly managed by the NHS, including private, public and hospital pharmacies, as well as local health units. All drugs were identified through the Anatomical Therapeutic Chemical index (ATC); the Co-payment Exemption Registry that collects information on individuals who benefit from disease-specific or income-based exemptions of co-payment of health care services.

The cohort consisted of all individuals diagnosed with lung cancer at the age of 18 or older between January 1, 2014 and December 31, 2017, and enrolled in the HAR at both the time of diagnosis (date of hospital discharge with a diagnosis of lung cancer) and during the preceding 5 years (“look-back” period). The look-back period aimed to focus on incident lung cancer cases, excluding patients with a previous diagnosis. Individuals with lung cancer diagnosis (both primary or secondary) were identified and retrieved from HIS using the following ICD-9-CM codes: 162.2, 162.3, 162.4, 162.5, 162.8, 162.9 [20, 21]. The date of the first diagnosis of lung cancer was defined as the index date. Patients were excluded from the cohort if any of the following conditions were detected in the look-back period: any cancer diagnosis (including lung cancer); receipt of one or more neoplastic drug treatments in the 12 months prior to the index date; or one or more exemption codes for oncological pathology (code 048). The overall population was then stratified by presence of metastases: metastatic (M) cohort (ICD-9-CM: 196, 197—excluding 197.0—and 198) [22, 23] and non-metastatic (NM) cohort.

### SEP indicators

The level of education, recorded in the HIS record, was used as a proxy for patients’ SEP. We considered this indicator as the primary measure of SEP for several reasons: it is a common approach used in public health research [24]; our study population consists predominantly of elderly individuals, where educational level strongly discriminates socioeconomic inequalities; and our research department has a long experience in socioeconomic inequality studies, using educational level as measure of SEP [25, 26]. SEP was classified into four groups based on educational level: low (none/elementary education), medium–low (middle school education), medium–high (high school education) and high (university education or more).

Further secondary potential indicators of SEP were used for sensitivity analyses:

1. the Italian deprivation index, based on 2011 census data, provides an indication at the geographically aggregated level of the municipality. This indicator is constructed from five variables: low level of education, unemployment, non-home ownership, one parent family and overcrowding [27, 28].
2. Income-based co-payment exemptions available in the Lazio database at the patient level (codes E01, E02, E03, E04). These exemptions are provided by healthcare authorities to ensure that individuals or families facing financial difficulties can still access necessary medical services without incurring significant financial burden from healthcare-related expenses.

### Patient characteristics and clinical outcomes

From the health administrative databases, a series of variables related to the patients were retrieved or calculated: age, sex, the volume of the annual lung interventions (lobectomy or lung resection ICD-9-CM codes: 32.4, 32.5, 32.9, 32.6, 32.3, 32.29) of the hospitals where patients were diagnosed ((none (0); low volume (1–100); high volume (> 100)), previous hospitalization with a diagnosis of Chronic Obstructive Pulmonary Disease (COPD) (ICD-9-CM codes: 491, 492 and 496) [29, 30], presence of comorbidities based on the Charlson Comorbidity Index (excluded COPD), and the number of specialist visits with pulmonologist during the year prior to index date. The following outcomes were investigated separately in NM and M cohorts:

- receiving a delayed diagnosis;
- access to advanced drug therapies for lung cancer within the two years following diagnosis;
- mortality within the two years following diagnosis (the primary outcome of the study).

In particular, patients were considered to have a delayed diagnosis if their first hospitalization for lung cancer was preceded by an emergency room admission. The group of lung cancer treatments considered for the analyses were defined as “advanced drug therapies” and are reported in Table S1. Advanced drug therapies included high-cost drug therapies with approved indication for lung cancer and novel drug therapies approved for lung cancer in concomitance with the study period (from 2013 to 2018). Some of the novel treatments also granted the innovative treatment status by the Italian Medicines Agency, based on therapeutic need, added therapeutic value and robustness of the scientific evidence of the drug [31].

### Statistical analyses

Qualitative variables were reported using frequencies (n) and percentages (%). Age, sex, specialist visits by pulmonologist, comorbidities and previous diagnosis of COPD were considered as potential confounders. To estimate the overall survival curves, patients were divided into groups according to SEP levels. The comparison of the different survival curves was calculated using the Kaplan–Meier estimator and were compared between groups using the Log-rank test pairwise comparisons. Multivariate models were implemented to investigate the association between SEP and study outcomes. Mortality and the risk of access to advanced drug therapies for lung cancer were measured throughout the follow-up period using Cox models and results were expressed as hazard ratio (HR) and the corresponding 95% confidence interval (CI). These two outcomes were measured as time-to-event, while in the case of use of advances drug therapies was used the meaning time until first treatment.

The probability of receiving a delayed diagnosis was measured at index date using logistic regression and results were expressed as odds ratio (OR) and 95% CI. Additionally, a Chi-Square test of association was performed between the primary SEP indicator and the secondary SEP indicators, and survival analyses were replicated using the secondary indicators.

## Results

### Demographic and clinical characteristics of the cohorts

**Fig. 1:**
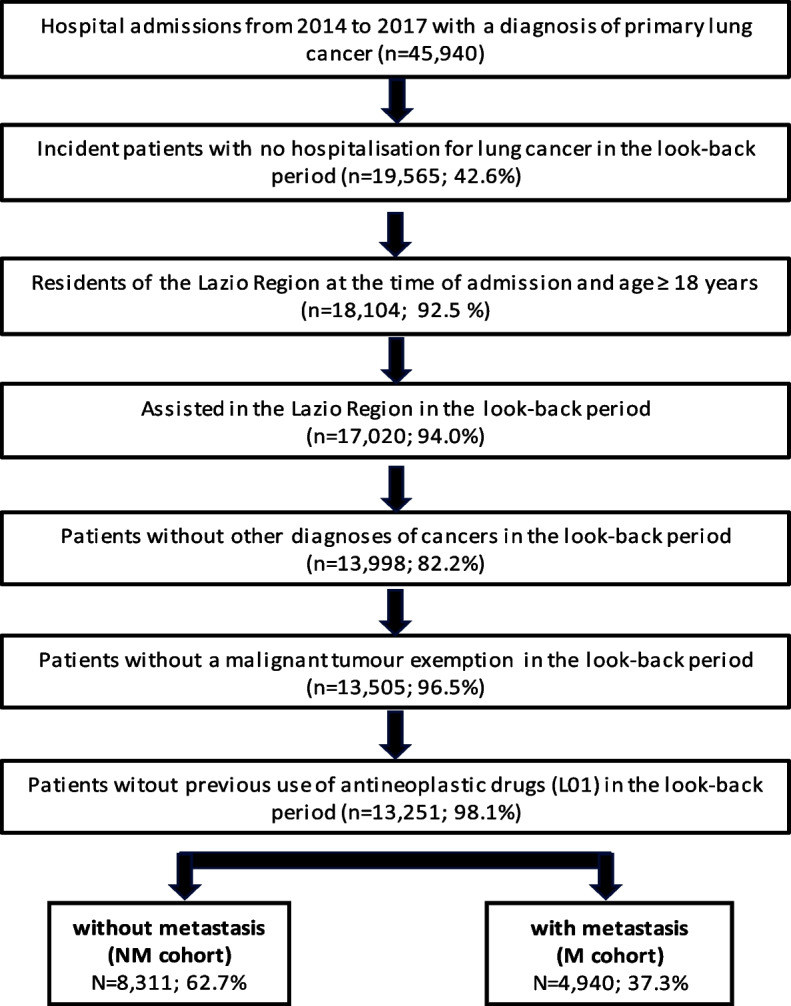
Flowchart of study population

In total, 13,251 incident cases of lung cancer were selected over the 4-year period (2014–2017) and included in the study. Out of these, 4,940 cases (37.3%) were included in the M cohort, while the remaining 8,311(62.7%) composed the NM cohort (Fig. 1). The study cohorts were separately described and their characteristics, both overall and stratified by SEP, are reported in Table 1. Among the two cohorts, similar characteristics were found: 63.0% and 64.4% of study participants, respectively in NM cohort and M cohorts, were male; 56.7% of NM and 50.7% of M patients were over 70 years of age and low percentages of both cohorts had 2 or more comorbidities according to Charlson Index (NM cohort: 5.9%, M cohort: 3.9%) even though moderate percentages of patients suffered from COPD (NM cohort: 10.1%, M cohort: 7.1%). Additionally, relative few from both cohorts visited a pulmonologist in the year before the index date (NM cohort: 29.8%, M cohort: 21.8%). Nearly half of patients from both cohorts (49.6% of NM and 40.6% of M) received lung cancer diagnosis from hospitals with a high volume of annual lung-related procedures.

**Table 1:** Demographic and clinic characteristics

	**Patients without metastasis (NM cohort)**										**Patients with metastasis (M cohort)**										**Overall**	
**SEP**	Low		Medium–low		Medium–high		High		Total		Low		Medium–low		Medium–high		High		Total		Total	
**N (%)**	2619	31.5	2364	28.4	2717	32.7	611	7.4	8311		1549	31.4	1450	29.4	1623	32.9	318	6.4	4940		13,251	
**Sex**																						
Male	1708	65.2	1372	58.0	1765	65.0	389	63.7	5234	63.0	1014	65.5	900	62.1	1073	66.1	195	61.3	3182	64.4	8416	63.5
Female	911	34.8	992	42.0	952	35.0	222	36.3	3077	37.0	535	34.5	550	37.9	550	33.9	123	38.7	1758	35.6	4835	36.5
**Age**																						
< = 60	112	4.3	483	17.8	563	23.8	131	21.4	1289	15.5	105	6.8	363	22.4	451	31.1	75	23.6	994	20.1	2283	17.2
61–70	453	17.3	863	31.8	791	33.5	198	32.4	2305	27.7	357	23.0	511	31.5	463	31.9	110	34.6	1441	29.2	3746	28.3
71–80	1126	43.0	958	35.3	739	31.3	196	32.1	3019	36.3	639	41.3	528	32.5	399	27.5	87	27.4	1653	33.5	4672	35.3
= > 80	928	35.4	413	15.2	271	11.5	86	14.1	1698	20.4	448	28.9	221	13.6	137	9.4	46	14.5	852	17.2	2550	19.2
**Charlson comorbidity index**																						
0	1785	68.2	2091	76,96	1938	82.0	516	84.5	6330	76.2	1195	77.2	1297	79.9	1221	84.2	281	88.4	3994	80.9	10,324	77.9
1	601	23.0	472	17.4	335	14.2	81	13.3	1489	17.9	276	17.8	259	16.0	185	12.8	32	10.1	752	15.2	2241	16.9
= > 2	233	8.9	154	5.7	91	3.9	14	2.3	492	5.9	78	5.0	67	4.1	44	3.0	5	1.6	194	3.9	686	5.2
**COPD**																						
No	2227	85.0	2440	89.8	2214	93.7	593	97.1	7474	89.9	1414	91.3	1490	91.8	1379	95.1	307	96.5	4590	92.9	12,064	91.0
Yes	392	15.0	277	10.2	150	6.3	18	2.9	837	10.1	135	8.7	133	8.2	71	4.9	11	3.5	350	7.1	1187	9.0
**Number of specialist visits with pulmonologist**																						
0	471	77.1	1675	70.9	1832	67.4	1856	70.9	5834	70.2	253	79.6	1129	77.9	1279	78.8	1204	77.7	3865	78.2	9699	73.2
1	50	8.2	217	9.2	285	10.5	240	9.2	792	9.5	19	6.0	133	9.2	138	8.5	131	8.5	421	8.5	1213	9.2
2	39	6.4	207	8.8	234	8.6	181	6.9	661	8.0	26	8.2	106	7.3	98	6.0	99	6.4	329	6.7	990	7.5
3	28	4.6	145	6.1	205	7.5	180	6.9	558	6.7	13	4.1	44	3.0	72	4.4	64	4.1	193	3.9	751	5.7
> 4	23	3.8	120	5.1	161	5.9	162	6.2	466	5.6	7	2.2	38	2.6	36	2.2	51	3.3	132	2.7	598	4.5
**Volume of lung interventions**																						
0	1020	38.9	648	23.8	316	13.4	67	11.0	2051	24.7	680	43.9	552	34.0	304	21.0	57	17.9	1593	32.2	3644	27.5
1–100	696	26.6	721	26.5	550	23.3	170	27.8	2137	25.7	464	30.0	467	28.8	329	22.7	80	25.2	1340	27.1	3477	26.2
> 100	903	34.5	1348	49.6	1498	63.4	374	61.2	4123	49.6	405	26.1	604	37.2	817	56.3	181	56.9	2007	40.6	6130	46.3

Regarding the main SEP indicator, similar distributions of patients were detected in both cohort: ~ 31% of patients had low SEP, ~ 29% had medium–low SEP, ~ 32% had medium–high SEP and ~ 7% had high SEP.

The results obtained from the stratification of demographic and clinical characteristics by SEP revealed that as SEP increased (from low to high), the percentages of patients with multiple comorbidities decreased from 11.8% to 3.3% in NM cohort and from 6.3% to 2.5% in M cohort. Similar phenomenon was observed for patients with COPD diagnosis, with a decrease from 15.0% to 2.9% in NM cohort and from 8.7% to 3.5% in M cohort. In addition, higher SEP was associated with younger age and admission at hospital with higher volume of annual lung-related procedures.

### Associations between SEP and clinical outcomes

**Fig. 2:**
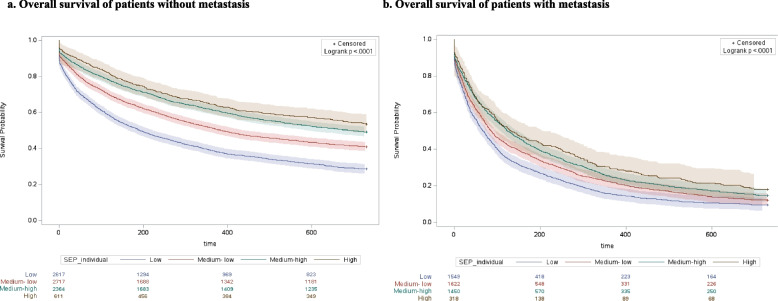
Overall survival of patients without metastasis (**a**) and patients with metastasis (**b**)

**Fig. 3:**
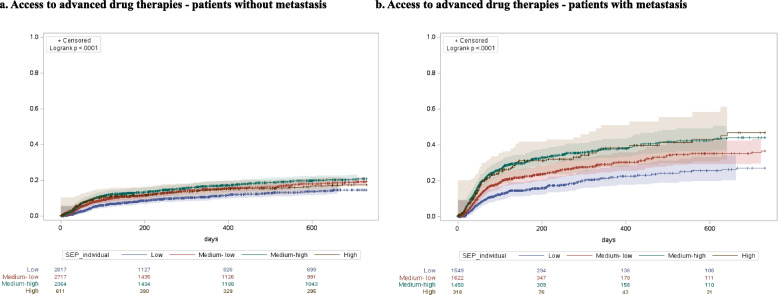
Access to advanced drug therapies of patients without metastasis (**a**) and patients with metastasis (**b**)

**Fig. 4:**
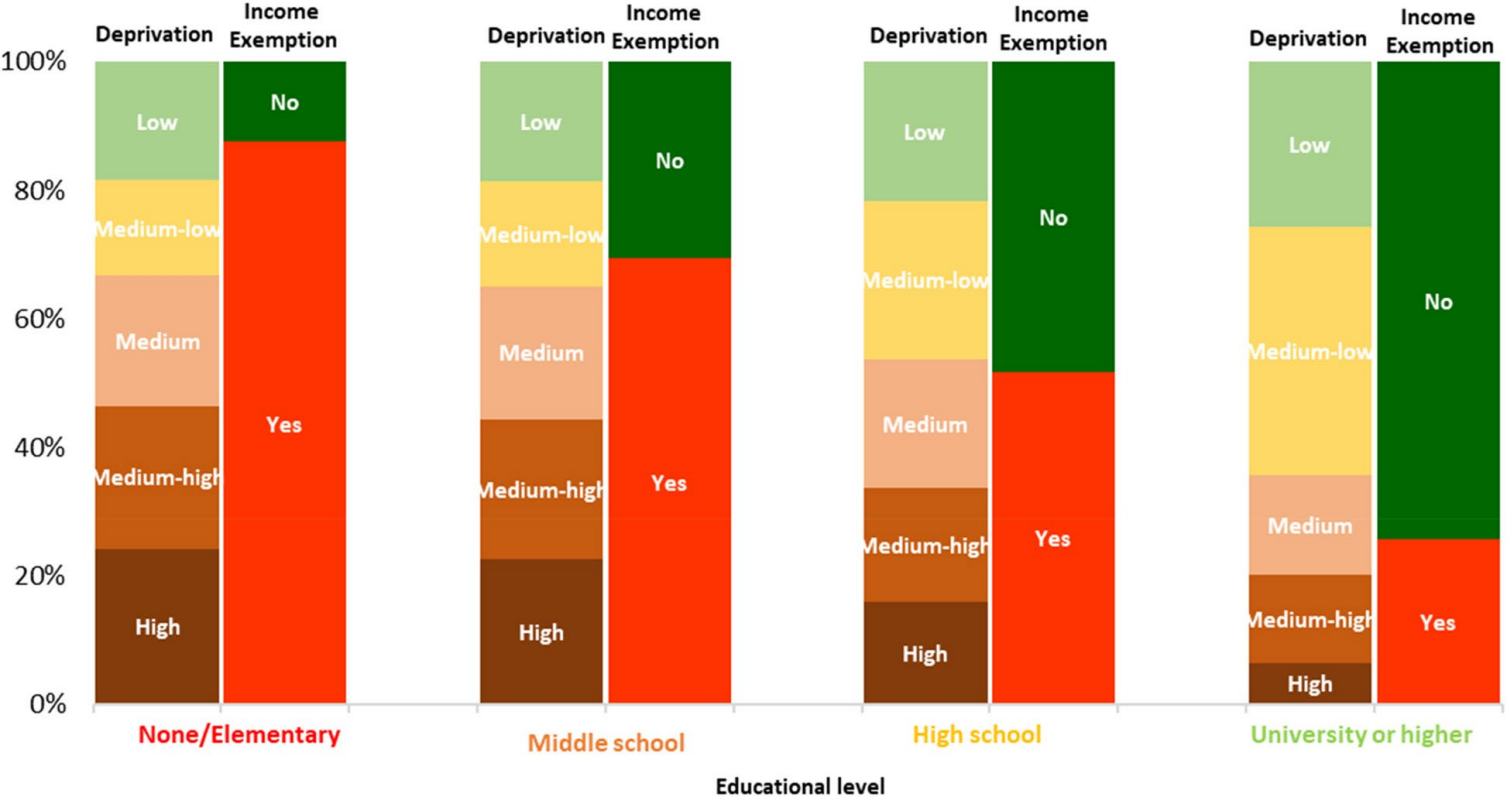
Association between SEP measures None/Elementary: low educational level; Middle School: medium–low educational level; High School: medium–high educational level; University or higher: high educational level. Color gradation from red to pink: educational level (from low to medium–low), deprivation (from high to medium) and presence of income exemption. Color gradation from yellow to green: educational level (from medium–high to high), deprivation (from medium–low to low) and absence of income exemption

**Fig. 5:**
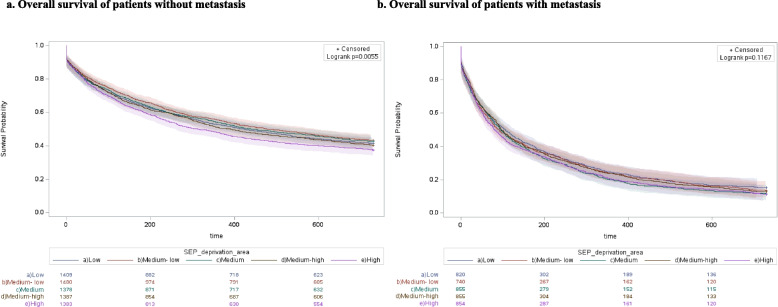
Level of deprivation and overall survival of patients without metastasis (**a**) and patients with metastasis (**b**)

**Fig. 6:**
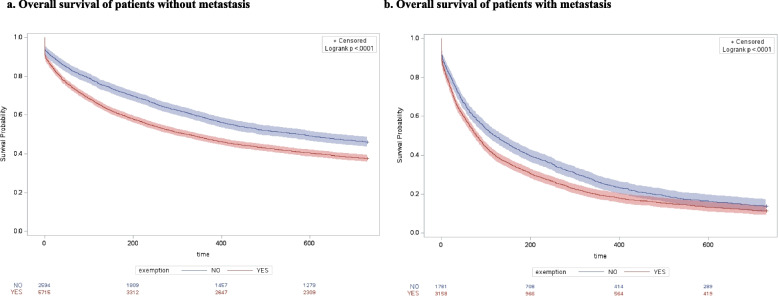
Income exemption and overall survival of patients without metastasis (**a**) and patients with metastasis (**b**)

The results of overall survival and access to advanced drug therapies, separated for M and NM cohorts and stratified by SEP, are represented in Figs. 2 and 3 respectively. In Fig. 2 at each time point, and in both cohorts, poorest survival was found in patients with low SEP, and results of the log-rank test detected a statically significantly difference between survival curves, both in NM (*p* < 0.0001) and M (*p* < 0.0001) cohorts. In Fig. 3 the log-rank test detected a statically significantly difference between curves, both in NM (*p* < 0.0001) and M (*p* < 0.0001) cohorts. However, the different trends observed in the curves of the M cohort, compared with those of the NM cohort, change over time and delineate higher access to advanced drug therapies among patients with high and middle SEP, compared with the others. Furthermore, there was a high association (*p* < 0.0001) between the primary (educational level) and the secondary (the level of deprivation and the income-based exemption) SEP measures considered in our study (Fig. 4). In addition, the curves obtained in the sensitivity analysis confirmed a trend in overall survival associated with SEP, particularly among NM patients (Figs. 5 and 6).

With respect to the outcomes under study, delayed diagnosis was found in 58.1% of M and 40.0% of NM patients (Table 2). According to multivariate analyses, as SEP increased, the risk of receiving a delayed diagnosis decreased in both cohorts. In particular, patients with high SEP had 70–80% reduced risk of receiving a delayed diagnosis compared to those with low SEP (NM: OR 0.20, 95% CI 0.16–0.25; M: OR 0.29, 95% CI 0.23–0.38). Access to advanced drug therapies for lung cancer was found in 16.4% of M patients and 12.2% of NM patients. We also calculated same proportions among patients receiving at least one prescription for antineoplastic agents (ATC: L01) and values increased as follow: 52.7% for NM and 56.2% for M patients. Further details on utilization of antineoplastic agents are provided in supplementary materials (Table S2).

**Table 2:** Association between SEP level and study outcomes, stratified by presence of metastases

	**NM cohort**			**M cohort**		
**Delayed diagnosis (N, %)**	3322	40.0%		2871	58.1%	
**SEP**	OR^a^	95% CI		OR^a^	95% CI	
Low (Ref)	1.00			1.00		
Medium–low	0.77	0.68	0.86	0.99	0.85	1.16
Medium–high	0.45	0.40	0.51	0.68	0.58	0.79
High	0.20	0.16	0.25	0.29	0.23	0.38
**Access to advanced drug therapies (N, %)**	1011	12.2%		810	16.4%	
**SEP**	HR^a^	95% CI		HR^a^	95% CI	
Low (Ref)	1.00			1.00		
Medium–low	1.01	0.85	1.21	1.17	0.96	1.44
Medium–high	1.07	0.89	1.28	1.54	1.26	1.88
High	0.90	0.69	1.16	1.57	1.18	2.09
**Mortality (N, %)**	4965	59.7%		4331	87.7%	
**SEP**	HR^a^	95% CI		HR^a^	95% CI	
Low (Ref)	1.00			1.00		
Medium–low	0.87	0.81	0.93	0.96	0.89	1.03
Medium–high	0.72	0.67	0.78	0.90	0.83	0.97
High	0.61	0.54	0.69	0.77	0.68	0.88

*NM* Non-metastatic, *M* Metastatic, *OR* Odds ratio, *HR* Hazard ratio, *CI* Confidence interval

^a^Adjusted for: Age, sex, referrals to pulmonologist and CCI

Results of multivariate analyses did not show significant differences in access based on SEP level, except for M patients with medium–high and high SEP compared to those with low SEP (medium–high: HR 1.54, 95% CI 1.26–1.88; high: HR 1.57, 95% CI 1.18–2.09). All-cause mortality rates were 87.7% for M and 59.7% for NM; mortality risk decreased steadily as SEP increased. Compared to patients with low SEP, those with high SEP had lower mortality risk: M: HR 0.77, 95% CI 0.68–0.88; NM: HR 0.61, 95% CI 0.54–0.69. This decreasing trend in mortality risk was more pronounced in NM cohort compared to M cohort (Table 2).

## Discussion

Our study highlights the impact of socioeconomic disparities on clinical outcomes among patients with M and NM lung cancer diagnosed and assisted in Lazio Region. Patients from both cohorts who were in poorer socio-economic conditions were more likely to experience delayed diagnosed and higher mortality rates within two years of diagnosis. Additionally, disparities in the access to advanced drug therapies for lung cancer were observed primarily in M patients.

Lung cancer patients of our cohort were predominantly elderly males (older than 70 years) and in line with previous evidence [32–35] approximately 40% of them presented metastasis at diagnosis. The considerable proportion of delayed diagnoses in our cohorts is in line with findings from a recent multinational study where, despite the huge variability found between countries, lung cancer patients were those most frequently diagnosed after emergency department admission [36]. Socioeconomically disadvantages patients are at higher risk of receiving their first diagnosis following in such emergency setting [37–39] and emergency department utilization among these patients involves all aspects of care, including diagnosis [40–42]. In general, although SEP may exacerbate the situation, the frequent delay of lung cancer diagnoses suggests that the disease is often identified at an advanced stage [4]. Although screening programs could potentially improve early detection rates Italy’s nationwide initiatives are still in early stage [43]. In addition, factors like as race and socioeconomic status may hinder access to these programs [14, 15]. In this context, patients in more favorable socioeconomic conditions are less likely to have COPD and other comorbidities compared to those more disadvantaged [13]. Even after adjusting for comorbidities, and age, residual confounding cannot be ruled out. Probably, a comprehensive approach, not only limited to the implementation of screening programs may be required to reduce later stage diagnosis in this population, including investments in healthcare and roadway infrastructures, the creation of effective prevention plans and the dissemination of educational campaigns able to reach the overall population regardless of the age and socioeconomic condition.

In terms of access to advanced drug therapies for lung cancer, the aim of our study was to ascertain whether in the two years following index date the use of advanced drug therapies might be influenced by the patient’s SEP. For these reasons we did not consider surgery, radiotherapy and traditional chemotherapy for the present analyses. Results showed that similar overall percentages of M and NM patients received at least one prescription during the follow-up period. Values increased by ~ 40.0 percentage points when proportions referred to patients receiving at least one prescription for antineoplastic agents. Differences in access to advanced drug therapies were only found among M patients, suggesting that socioeconomic disparities in this aspect are not widespread. This evidence does not completely reflect our expectations, given that the Italian healthcare system provides universal access to medical care, including therapies. The highly severe clinical condition of metastatic patients may have compelled those with higher SEP to use their socioeconomic resources to easier and faster access to treatments. We have also hypothesized that patients’ clinical characteristics may have contributed to these disparities. In particular, on one hand, treatment indications for the medications considered in our study are mostly limited to advanced patients with specific treatment history and clinical profiles, consequently some patients would have been excluded only because they were not eligible for these treatments. On the other hand, the worse prognosis of patients with low SEP both in terms of diagnosis and mortality, may have potentially reduced access to treatments because they died earlier compared to others. All these potential causes can be investigated in future studies involving more patients’ clinical characteristics that are not available on our administrative databases. Similar findings from nationwide studies conducted in countries with free access to anticancer therapies support our results. In details, M patients with lung cancer belonging to more deprived areas showed reduced utilization of innovative and novel therapies compared those living in more privileged areas [44, 45]. In addition, these findings also suggest that it would be of interest to investigate in the future differences between and within countries. Indeed, despite the fact that access to medical care in Italy should not present economic barriers, it faces several challenges related to the availability of care facilities, coordination, healthcare professionals, and equipment, with interregional differences [17].

Regarding mortality, a key focus of our study, previous research supports our evidence. National and international studies have shown significant social disparities among patients with advanced non-small cell lung cancer, based on educational level, income or area-level deprivation [46–51]. However, a study conducted in Piedmont, a region in Northern Italy, reported that survival in lung cancer patients was not associated with educational level both in early and advanced stage cancer [52], suggesting potential regional differences in patient management. Concerning the higher SEP gradient found among NM lung cancer patients compared to M patients, we hypothesized that the poorer clinical condition of advanced lung cancer patients may have mitigated the effect of socioeconomic inequalities on mortality, and our sensitivity analyses supported this trends. Overall, our study sheds light on socioeconomic inequalities existing among different components of the lung cancer care pathway in the Lazio region. These results provide evidence on a topic very little investigated in our country and indicate that future steps should to be taken to mitigate these disparities. For instance, in accordance with a recent literature review, the implementation of targeted populations interventions, such as those raising community awareness, or providing tailored sociocultural materials, or considering costs related to participation [53] may improve access to lung cancer screening in our country.

### Strengths and limitations

Our study has several strengths. Primarily, the observational nature of the study allowed the integration of many different types of healthcare administrative databases, that have long been used in different observational studies. These databases give the possibility to obtain precious results concerning epidemiological, effectiveness, and safety outcomes related to very large populations.

Secondly, we used individual measures of socioeconomic status, reducing biases related to aggregated measure. Furthermore, our analyses showed agreement between different SEP indicators and similar results in terms of association with mortality. Another strength of our study is that the focus of our study is in line with those of international and national projects: the 2030 Agenda Sustainable Development Goals adopted by the United Nations [54], the Cancer Inequalities Registry [55], a project realized by the European Commission with the aim of defining trends, disparities and inequalities between European countries and regions and the *Piano Oncologico Nazionale 2023–2027*, recently published by the Italian Ministry of Health with the aim of improve the primary, the secondary and the tertiary preventions in our country [56]. Further to the improvement of territorial health infrastructures and the implementation of lung cancer screening programmes in Italy, aspects already mentioned previously [17], our study emphasizes the importance of prevention strategies aiming to reduce the socioeconomic gap in this population. In particular, our work sheds light on the importance of education in this population of cancer patients, considering that this factor strongly influences awareness and knowledge regarding preventive and healthy behaviors [12, 57, 58].

However, our study has limitations. The lack of specific social and clinical parameters, including obesity and smoking habits, that are known to be strongly linked to education and mortality [12, 59, 60] and results from biopsy and pulmonary function tests (spirometry) that could affect the severity of disease and patient’s care-pathway. Moreover, as already mentioned, residual confounding may remain, both in terms of unmeasurable and incomplete variables (e.g., COPD), can't be avoided in the context of observational studies. A further limitation of our results is that they refer to one region only, despite Lazio represents a significant portion of Italy's population (10.0%) and receive patients from across the entire Italian territory [61–63].

Overall, future studies including additional clinical information, such as data retrieved from the Italian Cancer Registry, and involving more Italian regions are warranted, in order to improve limitations of our study and further investigate and foster knowledge on potential disparities existing among lung cancer patients.

## Conclusions

Our findings highlight the need for a more equitable and accessible healthcare management for lung cancer patients in Italy. Actions should be taken to improve care pathways, especially in terms of diagnosis and mortality. Further insights may better investigate and clarify the role of SEP on the access to treatments. Specific interventions, such as the implementation of educational programs, may increase the awareness and knowledge of this highly deadly disease before diagnosis, and promote equal utilization of services mitigating the effects of SEP on disease prognosis in this population.

### Supplementary Information

Supplementary Material 1.

## Data Availability

The data supporting the findings of this article are available at aggregated level from the authors upon reasonable request and with permission of Lazio region. Requests to access should be directed to Valeria Belleudi, Scientific Manager of the study, email: v.belleudi@deplazio.it.

## Abbreviations

SEP: Socioeconomic position
M: Metastatic
NM: Non-metastatic
OR: Odds ratio
HR: Hazard ratio
CI: Confidence interval
HAR: Healthcare Assistance Registry
HIS: Hospital Information System
NHS: National Health System
ICD-9-CM: International Classification of Diseases, 9th revision—Clinical Modification
COPD: Chronic Obstructive Pulmonary Disease
CCI: Charlson comorbidity index
NPR: Number of referrals to pulmonologist
VLI: Volume of lung interventions

## References

[1] Vos T, Lim SS, Abbafati C, Abbas KM, Abbasi M, Abbasifard M (2020). Global burden of 369 diseases and injuries in 204 countries and territories, 1990–2019: a systematic analysis for the Global Burden of Disease Study 2019. Lancet.

[2] Ferlay J, Colombet M, Soerjomataram I, Parkin DM, Piñeros M, Znaor A (2021). Cancer statistics for the year 2020: An overview. Int J Cancer.

[3] AIOM. I numeri del cancro in Italia. Intermedia. 2022. https://www.aiom.it/wp-content/uploads/2022/12/2022_AIOM_NDC-web.pdf. Accessed 14 Sep 2023.

[4] Malalasekera A, Nahm S, Blinman PL, Kao SC, Dhillon HM, Vardy JL (2018). How long is too long? A scoping review of health system delays in lung cancer. Eur Respir Rev.

[5] de Koning HJ, van der Aalst CM, de Jong PA, Scholten ET, Nackaerts K, Heuvelmans MA (2020). Reduced lung-cancer mortality with volume CT screening in a randomized trial. N Engl J Med.

[6] Pastorino U, Silva M, Sestini S, Sabia F, Boeri M, Cantarutti A (2019). Prolonged lung cancer screening reduced 10-year mortality in the MILD trial: new confirmation of lung cancer screening efficacy. Ann Oncol.

[7] National Lung Screening Trial Research Team (2019). Lung cancer incidence and mortality with extended follow-up in the National Lung Screening Trial. J Thorac Oncol.

[8] Burzic A, O’dowd EL, Baldwin DR (2022). The future of lung cancer screening: current challenges and research priorities. Cancer Manag Res.

[9] Tyczynski JE, Bray F, Parkin DM (2003). Lung cancer in Europe in 2000: epidemiology, prevention, and early detection. Lancet Oncol.

[10] Malhotra J, Malvezzi M, Negri E, La Vecchia C, Boffetta P (2016). Risk factors for lung cancer worldwide. Eur Respir J.

[11] Thandra KC, Barsouk A, Saginala K, Aluru JS, Barsouk A (2021). Epidemiology of lung cancer. Wspolczesna Onkologia.

[12] Braveman P, Gottlieb L (2014). The social determinants of health: it’s time to consider the causes of the causes. Public Health Rep.

[13] McMaughan DJ, Oloruntoba O, Smith ML (2020). Socioeconomic status and access to healthcare: interrelated drivers for healthy aging. Frontiers.

[14] Sosa E, D’Souza G, Akhtar A, Sur M, Love K, Duffels J (2021). Racial and socioeconomic disparities in lung cancer screening in the United States: a systematic review. CA Cancer J Clin.

[15] Castro S, Sosa E, Lozano V, Akhtar A, Love K, Duffels J (2021). The impact of income and education on lung cancer screening utilization, eligibility, and outcomes: a narrative review of socioeconomic disparities in lung cancer screening. J Thorac Dis.

[16] Singh GK, Jemal A (2017). Socioeconomic and racial/ethnic disparities in cancer mortality, incidence, and survival in the United States, 1950–2014: over six decades of changing patterns and widening inequalities. J Environ Public Health.

[17] OECD. EU country cancer profile: Italy 2023. EU country cancer profiles. Paris: OECD Publishing; 2023. 10.1787/a0a66c1d-en.

[18] Zhang X, Elsaid MI, DeGraffinreid C, Champion VL, Paskett ED, Brock G (2023). Impact of the COVID-19 pandemic on cancer screening delays. J Clin Oncol.

[19] Muka T, Li JJ, Farahani SJ, Pa Ioannidis J. An umbrella review of systematic reviews on the impact of the COVID-19 pandemic on cancer prevention and management, and patient needs. Elife. 2023;12:85679. 10.7554/eLife.85679 PMC10156163 37014058

[20] Cortinovis DL, Perrone V, Giacomini E, Sangiorgi D, Andretta M, Bartolini F (2023). Epidemiology, patients' journey and healthcare costs in early-stage non-small-cell lung carcinoma: a real-world evidence analysis in Italy. Pharmaceuticals (Basel).

[21] Montedori A, Bidoli E, Serraino D, Fusco M, Giovannini G, Casucci P (2018). Accuracy of lung cancer ICD-9-CM codes in Umbria, Napoli 3 Sud and Friuli Venezia Giulia administrative healthcare databases: a diagnostic accuracy study. BMJ Open.

[22] Franchi M, Rea F, Santucci C, La Vecchia C, Boffetta P, Corrao G (2021). Developing a multimorbidity prognostic score in elderly patients with solid cancer using administrative databases from Italy. Aging Cancer.

[23] MacLean E, Louder A, Saverno K, Smith G, Mardekian J, Brunis C (2016). Molecular testing patterns in metastatic non-small cell lung cancer. Am J Manag Care.

[24] Braveman PA, Cubbin C, Egerter S, Williams DR, Pamuk E (2010). Socioeconomic disparities in health in the United States: what the patterns tell us. Am J Public Health.

[25] Ventura M, Mataloni F, Colais P, Davoli M (2019). Fusco D [More equity in Lazio region health care: results from the Regional Outcome Evaluation Program (PReValE), 2012–2017]. Epidemiol Prev.

[26] Dipartimento di Epidemiologia del Servizio Sanitario Regionale del Lazio. Salute ed equità nella Regione Lazio. Roma: Il pensiero Scientifico Editore; 2022.

[27] Caranci N, Biggeri A, Grisotto L, Pacelli B, Spadea T (2010). Costa G [The Italian deprivation index at census block level: definition, description and association with general mortality]. Epidemiol Prev.

[28] Rosano A, Pacelli B, Zengarini N, Costa G, Cislaghi C (2020). Caranci N [Update and review of the 2011 Italian deprivation index calculated at the census section level]. Epidemiol Prev.

[29] Cazzola M, Puxeddu E, Bettoncelli G, Novelli L, Segreti A, Cricelli C (2011). The prevalence of asthma and COPD in Italy: a practice-based study. Respir Med.

[30] Shaya FT, Dongyi D, Akazawa MO, Blanchette CM, Wang J, Mapel DW (2008). Burden of concomitant asthma and COPD in a Medicaid population. Chest.

[31] AIFA. Innovative medicinal products. 2016. https://www.aifa.gov.it/en/farmaci-innovativi. Accessed 14 Sept 2023.

[32] Matsuda A, Matsuda T, Shibata A, Katanoda K, Sobue T, Nishimoto H (2014). Cancer incidence and incidence rates in Japan in 2008: a study of 25 population-based cancer registries for the Monitoring of Cancer Incidence in Japan (MCIJ) project. Jpn J Clin Oncol.

[33] Little AG, Gay EG, Gaspar LE, Stewart AK (2007). National survey of non-small cell lung cancer in the United States: epidemiology, pathology and patterns of care. Lung Cancer.

[34] Morgensztern D, Ng SH, Gao F, Govindan R (2010). Trends in stage distribution for patients with non-small cell lung cancer: a National Cancer Database survey. J Thorac Oncol.

[35] Tamura T, Kurishima K, Nakazawa K, Kagohashi K, Ishikawa H, Satoh H (2015). Specific organ metastases and survival in metastatic non-small-cell lung cancer. Mol Clin Oncol.

[36] McPhail S, Swann R, Johnson SA, Barclay ME, Abd Elkader H, Alvi R (2022). Risk factors and prognostic implications of diagnosis of cancer within 30 days after an emergency hospital admission (emergency presentation): an International Cancer Benchmarking Partnership (ICBP) population-based study. Lancet Oncol.

[37] Pollock AM, Vickers N (1998). Deprivation and emergency admissions for cancers of colorectum, lung, and breast in south east England: ecological study. BMJ.

[38] Beckett P, Tata LJ, Hubbard RB (2014). Risk factors and survival outcome for non-elective referral in non-small cell lung cancer patients - analysis based on the National Lung Cancer Audit. Lung Cancer.

[39] Raine R, Wong W, Scholes S, Ashton C, Obichere A, Ambler G (2010). Social variations in access to hospital care for patients with colorectal, breast, and lung cancer between 1999 and 2006: Retrospective analysis of hospital episode statistics. BMJ.

[40] Zhou Y, Abel GA, Hamilton W, Pritchard-Jones K, Gross CP, Walter FM (2016). Diagnosis of cancer as an emergency: a critical review of current evidence. Nat Rev Clin Oncol.

[41] Meert AP, Sculier JP, Berghmans T (2015). Lung cancer diagnosis in the emergency department. Eur Respir J.

[42] Walder JR, Faiz SA, Sandoval M (2023). Lung cancer in the emergency department. Emerg Cancer Care.

[43] Istituto Nazionale dei Tumori. Rete Italiana Screening Polmonare (RISP). 2022. https://programmarisp.it/. Accessed 25 Oct 2023.

[44] Scherpereel A, Durand-Zaleski I, Cotté FE, Fernandes J, Debieuvre D, Blein C (2018). Access to innovative drugs for metastatic lung cancer treatment in a French nationwide cohort: the TERRITOIRE study. BMC Cancer.

[45] Norris RP, Dew R, Greystoke A, Todd A, Sharp L (2023). Socioeconomic inequalities in novel NSCLC treatments during the era of tumor biomarker-guided therapy: a population-based cohort study in a publicly funded health care system. J Thorac Oncol.

[46] Di Maio M, Signoriello S, Morabito A, Rossi A, Maione P, Piantedosi FV (2012). Prognostic impact of education level of patients with advanced non-small cell lung cancer enrolled in clinical trials. Lung Cancer.

[47] Hussain SK, Lenner P, Sundquist J, Hemminki K (2008). Influence of education level on cancer survival in Sweden. Ann Oncol.

[48] Johnson AM, Hines RB, Johnson JA, Bayakly AR (2014). Treatment and survival disparities in lung cancer: the effect of social environment and place of residence. Lung Cancer.

[49] Chang CM, Su YC, Lai NS, Huang KY, Chien SH, Chang YH (2012). The combined effect of individual and neighborhood socioeconomic status on cancer survival rates. PLoS One.

[50] Finke I, Behrens G, Schwettmann L, Gerken M, Pritzkuleit R, Holleczek B (2020). Socioeconomic differences and lung cancer survival in Germany: Investigation based on population-based clinical cancer registration. Lung Cancer.

[51] Chouaïd C, Debieuvre D, Durand-Zaleski I, Fernandes J, Scherpereel A, Westeel V (2017). Survival inequalities in patients with lung cancer in France: A nationwide cohort study (the TERRITOIRE Study). PLoS One.

[52] Pagano E, Filippini C, Di Cuonzo D, Ruffini E, Zanetti R, Rosso S (2010). Factors affecting pattern of care and survival in a population-based cohort of non-small-cell lung cancer incident cases. Cancer Epidemiol.

[53] Sayani A, Ali MA, Dey P, Corrado AM, Ziegler C, Nicholson E (2023). Interventions designed to increase the uptake of lung cancer screening: an equity-oriented scoping review. JTO Clin Res Rep.

[54] UN General Assembly. Transforming our world : the 2030 Agenda for Sustainable Development, 2015, A/RES/70/1. https://www.refworld.org/docid/57b6e3e44.html. Accessed 14 Sep 2023.

[55] European Commission. European cancer inequalities registry. 2021. https://cancer-inequalities.jrc.ec.europa.eu/. Accessed 14 Sep 2023.

[56] Ministero della Salute. Piano Oncologico Nazionale: documento di pianificazione e indirizzo per la prevenzione e il contrasto del cancro 2023–2027. https://www.salute.gov.it/imgs/C_17_pubblicazioni_3291_allegato.pdf. Accessed 14 Sep 2023.

[57] Pampel FC, Krueger PM, Denney JT (2010). Socioeconomic disparities in health behaviors. Annu Rev Sociol.

[58] Zajacova A, Lawrence EM (2018). The relationship between education and health: reducing disparities through a contextual approach. Annu Rev Public Health.

[59] Huisman M, Kunst AE, Mackenbach JP (2005). Inequalities in the prevalence of smoking in the European Union: comparing education and income. Prev Med (Baltim).

[60] Egerter P, Braveman P, Sadegh-Nobari T, Grossman-Kahn R, Dekker M (2011). Education and health. Exploring the social determinants of health.

[61] Nante N, Messina G, Lispi L, Serafini A, Prisco G, Moirano F (2016). Mobility trends of patients across Italian Regions: implications for planning and evaluation of hospital services. Ann Ig.

[62] Nante N, Guarducci G, Lorenzini C, Messina G, Carle F, Carbone S (2021). Inter-regional hospital patients’ mobility in Italy. Healthcare (Switzerland).

[63] Balia S, Brau R, Marrocu E (2018). Interregional patient mobility in a decentralized healthcare system. Reg Stud.

