# Supplementary material for: Investigating socioeconomic disparities in lung cancer diagnosis, treatment and mortality: an Italian cohort study
Source: BMC Public Health. 2024 Jun 7;24:1543. doi: 10.1186/s12889-024-19041-4 (PMC11161996; doi:10.1186/s12889-024-19041-4)
Supplement: Supplementary file 1 — Supplementary Material 1. [file 12889_2024_19041_MOESM1_ESM.docx]

**Supplementary materials**

**Table S1. List of advanced drug therapies for lung cancer**

| **ATC code** | **Name** | **AIFA Approval date*** | **AIFA Innovative treatment criteria granted** |
| --- | --- | --- | --- |
| L01EB02 | erlotinib | July 2006 | No |
| L01XC07 | bevacizumab | June 2008 | No |
| L01CE01 or L01XX17 | topotecan | November 2008 | No |
| L01BA04 | pemetrexed | April 2009 | No |
| L01EB01 | gefitinib | May 2010 | No |
| L01CD01 | nab-paclitaxel | March 2014 | No |
| L01EB03 | afatinib | December 2014 | No |
| L01ED01 | crizotinib | April 2013 | March 2015 |
| L01XC17 | nivolumab | February 2017 | February 2017 |
| L01ED02 | ceritinib | June 2017 | No |
| L01XC18 | pembrolizumab | June 2017 | June 2017 |
| L01EB04 | osimertinib | August 2017 | No |
| L01ED03 | alectinib | July 2018 | July 2018 |
| L01XC32 | atezolizumab | July 2018 | July 2018 |

* Indication for Lung Cancer

**Table S2. List of the 5 most frequent antineoplastic agents not included in the “advanced drug therapy” outcome**

| **Metastatic patients (N=1441)** | | | |  | **Non metastatic patients (N=1920)** | | | |
| --- | --- | --- | --- | --- | --- | --- | --- | --- |
| **ATC** | **Active substance** | **n** | **%** |  | **ATC** | **Active substance** | **n** | **%** |
| L01XA02 | CARBOPLATIN | 271 | 18.8% |  | L01CA04 | VINORELBINE BITARTRATE | 354 | 18.4% |
| L01XA01 | CISPLATIN | 193 | 13.4% |  | L01XA02 | CARBOPLATIN | 289 | 15.1% |
| L01CA04 | VINORELBINE BITARTRATE | 175 | 12.1% |  | L01XA01 | CISPLATIN | 284 | 14.8% |
| L01BC05 | GEMCITABINE HYDROCHLORIDE | 134 | 9.3% |  | L01BC05 | GEMCITABINE HYDROCHLORIDE | 226 | 11.8% |
| L01CB01 | ETOPOSIDE | 103 | 7.1% |  | L01CB01 | ETOPOSIDE | 99 | 5.2% |
